# Supplementary material for: Relationship between true digestibility of dietary phosphorus and gastrointestinal bacteria of goats
Source: PLoS One. 2020 May 22;15(5):e0225018. doi: 10.1371/journal.pone.0225018 (PMC7244181; doi:10.1371/journal.pone.0225018)
Supplement: S1 Table — (PDF) [file pone.0225018.s001.pdf]

Table S1 The numbers of reads and OTUs

Taxa		HP.R	LP.R	HP.A	LP.A	HP.J	LP.J	HP.Co	LP.Co	HP.Ce	LP.Ce
Reads	**Total**	468063	445333	473295	456525	451392	447860	463396	443213	430165	381404
	**Average**	78160±6239	74353±3435	79014±6952	76229±7791	75340±9523	74774±5794	77453±3658	74056±6956	76766±4345	71854±3599
OTUs	**Total**	3608	4037	3850	3720	3977	3830	5654	5299	4402	4629
	**Average**	2052±74	2246±81	2086±186	2178±105	2313±179	2085±271	3052±374	2848±255	2609±358	2948±163
