# Supplementary material for: Relationship between true digestibility of dietary phosphorus and gastrointestinal bacteria of goats
Source: PLoS One. 2020 May 22;15(5):e0225018. doi: 10.1371/journal.pone.0225018 (PMC7244181; doi:10.1371/journal.pone.0225018)
Supplement: Attachment — Submitted filename: Response to reviewers.docx [file pone.0225018.s002.docx]

**Point-by-point Responses**

We greatly appreciate the insightful and careful review by the editor and reviewers and have made the following major revisions:

**Responses to editor:**

We checked our manuscript and corrected language errors carefully, and formatted our manuscript as requested.

Editor: Please ensure that your manuscript meets PLOS ONE's style requirements, including those for file naming.

**Response:We download the PLOS ONE style templates from the website, and formatted our manuscript as requested.**

Editor: In your Methods section, please provide additional information on the animal research and ensure you have included details on: (1) methods of blood collection, e.g. volume, location from which samples were taken, methods of anesthesia if relevant (2) methods of goat sacrifice, and (3) efforts to alleviate suffering.

**Response: we have revised our manuscript and the related contents were presented in L105-112 as “The next day after the end of the metabolism experiment, 10 ml of blood was collected from the jugular vein of each goat using a disposable vacuum blood collection tube before feeding in the morning.** **After anticoagulation, the plasma was collected and stored at -20 °C until use. Then the goats were slaughtered****. Pre-harvest handling was conducted in accordance with good animal welfare practices, and** **slaughtering procedures followed actual chinese law on animal production and sanitary inspection.”**

Editor: In your Data Availability statement, you have not specified where the minimal data set underlying the results described in your manuscript can be found. PLOS defines a study's minimal data set as the underlying data used to reach the conclusions drawn in the manuscript and any additional data required to replicate the reported study findings in their entirety. All PLOS journals require that the minimal data set be made fully available.

**Response:** In the “Data Availability Statement”, we state that “All the data of sequence in current research were placed in NCBI's sequence read archive (SRA) database with the accession No. SRP185613.”

Editor: PLOS requires an ORCID iD for the corresponding author in Editorial Manager on papers submitted after December 6th, 2016. Please ensure that you have an ORCID iD and that it is validated in Editorial Manager.

**Response:** The ORCID iD for Lizhi Wang is :https://orcid.org/0000-0003-3074-4273, which has been authenticated in Editorial Manager.

**point-by-point responses to the reviewers**

**Responses to Reviewer1:**

Two major comments have to be given:

**Reviewer1: the differences in dietary P supply were marginal , i. e., according to plasma Ca and P concentrations the dietary treatment cannot be assigned as "LP".**

**Response: We are so sorry, probably because our presentation is not clear enough, the reviewer did not fully understand the way of animal grouping. Please allow us to explain it here.**

**In the present study, the “LP” means low true digestibility of phosphorus rather than low dietary P level. The** **true digestibility of dietary phosphorus was determined by a two-stage** **metabolic experiment** **according to the Difference Level Technique (DLT). The dietary levels of phosphorus in the first and second** **metabolic experiments were 2.2 and 3.3 g/kg, respectively. During the two metabolic experiments, all of the 28 goats were fed the same diet, and then their** **true digestibility of dietary phosphorus was calculated according to DLT. According to the true digestibility of dietary phosphorus, the 28 goats were grouped into HP, MP and LP group.**

**Reviewer1: The whole study is not hypothesis driven. Thus, a clear concept is lacking.**

**Response: In fact, the hypothesis driving our study is implicit in the introduction, except that it is not clearly stated. According to your comment, to highlight the hypothesis of this study, we revised the our manuscript in L70-73 as “Therefore, in this study, we hypothesized that the digestibility of feed phosphorus in ruminants was influenced by the structure and composition of their gastrointestinal microbiota, and the 16S rRNA high-throughput sequencing technology was used to compare the bacterial diversity in the GIT of goats with different** **true digestibility of phosphorus (TDP).”.**

**Responses to Reviewer2:**

**Reviewer 2:** L28.phyla

**Response: We have revised it in L29.**

**Reviewer 2:** L49.paying to surface water eutrophication in parts where the livestock production of monogastric is intensive [2, 3] .Phytase a type of phosphatase enzyme that catalyzes….

**Response: we have revised in L50 as “contributing to surface water eutrophication in locations where there is intensive monogastric livestock production [2, 3]. Phytase is an enzyme that…”**

**Reviewer 2:** L67.technology has not been able to complete this work. In view of this fact, in this study the 16S rRNA….

**Response:****we have revised in L69 as“Therefore, in this study the 16S rRNA….”**

**Reviewer 2:** L69.true digestibility of phosphorus (TDP)….

**Response:we have revised in L67 as “true digestibilities of phosphorus….’’**

**Reviewer 2: L71. screening to identify high-efficiency.**

**Response:****we have revised in L71 as “screening to identify high-efficiency….”**

**Reviewer 2: L80. old healthy Nubian black goats with an average….**

**Response:we have revised in L80 as“old female** **healthy Nubian black goats with an average…”**

**Reviewer 2:** L82.All experimental goats were kept in pens (individual) with free choice to water.

**Response:we have revised in L82 as “Goats were kept in** **individual pens with free access to water”.**

**Reviewer 2:** L84. …presented in (Table 1) at two times at 8:00 am and 5:00 pm.

**Response:we have revised in L84 as “****as shown in (Table 1) fed two times…”**

**Reviewer 2: L86. delete sentence starting with The formulas**

**Response: we have delete sentence starting with the formulas.**

**Reviewer 2:** L90.VA: 2 937 IU, VD: 343 IU, VE: 30 IU.

**Response: we have revised in L90 as“****Vitamin A, Vitamin D and Vitamin E”**

**Reviewer 2: L97 collected. Was urine collected into acid? Or just mixed with acid after sampling?**

**Response: During the metabolism trial period, all the urine and feces were collected each day, and the feces and urine were sampled. The samples were mixed with hydrochloride.**

**Reviewer 2:** L100.the 6-days metabolism

**Response: we have revised in L100 as“6-day metabolism”**

**Reviewer 2:** L103.the GIT organs (rumen, abomasum, jeujinu, cecum and colon) were unglued to collect rumen (8 tubes, each of 50 mL), abomasums (4 tubes, each 15 mL), jejunum (4 tubes, each 15 mL), cecum (4 tubes, each 15 mL) and colon (4 tubes, each 15 mL) content .

**Response: we have revised inL103 as“jejunum…)** **were opened and the digesta was mixed and collected into tubes: rumen (8 tubes of 50 ml) ….”**

**Reviewer 2:** L105. A tube of ruminal sample was subjected to measure the pH (PHS…

**Response: we have revised in L105 as“****Ruminal sample pH was measured (PHS….”**

**Reviewer 2:** L107**.** **Ruminal fluid was strained through four layers of cheese cloth and 20 ml were mixed with 4 mL of 25%? Meta-phosphoric acid…
110 Samples from the GIT segments were stored at -80oC for further analysis.**

**Response:We have revised in L117 as “Subsequently, this rumen fluid (approximately 20 mL) was mixed with 5mL solution of 25% metaphosphoric acid and preserved at -20°C for further calculation of the volatile fatty acid concentration (VFA; acetate, propionate, and butyrate).”**

**Reviewer 2:** L110.The other remnants of GIT segment samples were stored at -80°C for further assessment.

**Response: we have revised in L110 as“****Samples from the GIT segments were stored at -80℃ for further analysis.”**

**Reviewer 2: L116…. gas chromatography**
**Response: we have revised in L116 as“gas chromatography”**

**Reviewer 2: L119. ground to pass a 40-mesh sieve**
**Response: we have revised in L119 as“ground to pass a 40-mesh sieve”**

**Reviewer 2:** L122. measure organic matter…crude ash. Neutral detergent fiber…What about mentioning specific procedure from AOAC book?

**Response: we have revised in L134-135 as “Neutral detergent fiber (NDF) and acid detergent fiber (ADF) were measured according to the procedures of AOAC (method NO. 973.18 C, 1990).”**

**Reviewer 2:** L124…..with the filter bag technique lacking of sodium sulfite and articulated with ash residual.

**Response:** **we have revised in L134-138.**

**Reviewer 2:** L136. True digestibility

**Response: we have revised in L136 as “true digestibility”**

**Reviewer 2: L142.** **TDP of all goats were calculated with mean…
Response: we have revised in L142 as “****TDP of all goats were calculated with mean…”**

**Reviewer 2: L142. Animals were grouped into high digestibility of phosphorus….**

**Response: we have revised in L142 as“****Animals were grouped into high digestibility of phosphorus….”**

**Reviewer 2: L147. and sterile phosphate buffered saline (pH 7) was used to rinse the liquid through 4 layers of cheesecloth and collected into a sterile tube (EP). The resulting liquid was immediately centrifuged at 10,000 x g and the supernatant gently removed.**

**Response: we have revised in L147 as“and sterile phosphate buffered saline (pH 7) was used to rinse the liquid through 4 layers of cheesecloth and collected into a sterile tube (EP). The resulting liquid was immediately centrifuged at 10,000 x g and the supernatant gently removed.”**

**Reviewer 2:** L152. **used for DNA extraction from samples of ruminal and homogenized digesta from other organs following the manufacturers instructions using the procedure described in [19]**

**Response: we have revised in L152 as “****used for DNA extraction from samples of ruminal and homogenized digesta from other organs following the manufacturers instructions using the procedure described in [19]”**

**Reviewer 2:** L154. **agrose electrophoresis and NanDrop 8000 spectrophotometer….**

**Response: we have revised in L154 as “****Agarose electrophoresis and NanoDrop….”**

**Reviewer 2:** L155….. **measure DNA quality of the samples**.

**Response: we have revised in L155 as “****measure** **DNA quality of the samples.”**

**Reviewer 2:** L184…..SILVA database….

**Response: we have revised in L184 as “SILVA”**

**Reviewer 2:** L204. The TDP of the 28 goats highly varied (from 68.38% to 90.26%), and the average was 83.28% ± 6.81%.

**Response: we have revised in L204 as “****The TDP of the goats varied from 68.4% to 90.3% with an average of 83.3% (± 6.8%).”**

**Reviewer 2: L205.** **The TDP of the 8 animals in the HP group 89.5% ± 2.9%) was significantly higher (than for the LP group (78.4% ± 1.21%).**

**Response: we have revised in L229-230 as “The TDP of the 8 animals in the HP group (89.5% ± 2.9%) was significantly higher than the LP group (78.4% ± 1.21%).”**

**Reviewer 2:** L209. **was significantly higher (P<0.05) than the LP group**),….

**Response: we have revised in L209 as“****was significantly higher (P<0.05) than the LP group”**

**Reviewer 2:** L209….. **not significantly different (P>0.05)**….

**Response: we have revised in L209 as “****not significantly different (P>0.05)”**

**Reviewer 2:** L230-359 While I recognize some phyla and genus, I am not familiar enough with literature to give a critical review. Yes, I have seen these in other research, but not knowledgeable enough to evaluate the research.

**Response: thank you.**

**Reviewer 2:** L368.what is the feeding standard recommendation?

**Response: The recommended phosphorus level of the Chinese meat sheep and goat feeding standard (NY/T 816-2004) is** **4.0-5.0 g/kg DMI.** **In the present study, the phosphorus** **dietary levels in the first and second metabolic experiments were 2.2 and 3.3 g/kg, respectively. We have revised in L394 as “…Chinese meat sheep and goat feeding standard (****4.0-5.0 g/kg DMI, NY/T 816-2004)”.**

**Reviewer 2:** L379. …. **goats varied greatly** ….

**Response: we have revised in L379 as “****goats** **varied greatly.”**

**Reviewer 2:** L382**…but prior to our research, there was no published report on the variation of TDP….**

**Response: we have revised in L382 as “****but prior to our research, there was no published report on the variation of TDP…”**

**Reviewer 2:** L383…. **earlier research showed that individual variations in feed efficiency of ruminants are common** [35-37].

**Response: we have revised in L383 as “****earlier research showed that individual variations in feed efficiency of ruminants are common […”**

**Reviewer 2:** L393. **microbial digestion is an important component of digestion in the…**

**Response: we have revised in L393 as “****microbial digestion is an important component of digestion in the…”**

**Reviewer 2:** L404.…. **yet been reported**.

**Response: we have revised in L404 as “****yet been** **reported.”**

**Reviewer 2:** L405…. **the binding of free phosphorus with some substance in feed such as protein…**…

**Response: we have revised in L405 as “****the binding of free phosphorus with some substance in feed such as protein…”**

**Reviewer 2: L421…nutrient availability…**

**Response:** **we have revised in L421 as “****nutrient availability”**

**Reviewer 2: 445** **CABI**

**Response:** **we have revised in L485 as “CABI”**

**Reviewer 2: 451 feeding**

**Response: we have revised in L491 as “feeding”.**

**Reviewer 2: 504** **excretion in**

**Response: we have revised in L540 as “excretion in ruminants”**

**Reviewer 2:** Why not in figure 1B and 3a CHANGE SYMBOLS. Use a solid square for HP rumen and an open square for LP rumen. Use a solid triangle for the abomasum HP and an open triangle for LP. Use a solid and open circle for jejunum and open and closed diamond for the colon.

**Response: Sorry, we can't revise our article as you suggested.**
