# Supplementary material for: Relationship between true digestibility of dietary phosphorus and gastrointestinal bacteria of goats
Source: PLoS One. 2020 May 22;15(5):e0225018. doi: 10.1371/journal.pone.0225018 (PMC7244181; doi:10.1371/journal.pone.0225018)
Supplement: Attachment — Submitted filename: Response to reviewers -.docx [file pone.0225018.s003.docx]

**Point-by-point Responses**

We greatly appreciate the insightful and careful review by the editor and reviewers and have made the following revisions.

**Responses to Comments**

**Comments: -how and where the goats were slaughtered**

**-the method of anaesthesia or analgesia used, if relevant**

**Response:** We have revised as per suggestion thanks.

The goats were slaughtered at slaughter house in Teaching and Research Base, Animal Nutrition Institute, Sichuan Agricultural University. All the goats were slaughter according to the American Veterinary Medical Association (AVMA) guidelines for the humane slaughter of animals.

Note: (The animals were stunned by captive bolt and the exsanguination from the jugular vein was carried out)
